# Chitosan Treatment of E-11 Cells Modulates Transcription of Nonspecific Immune Genes and Reduces Nodavirus Capsid Protein Gene Expression

**DOI:** 10.3390/ani11113097

**Published:** 2021-10-29

**Authors:** Nadia Chérif, Fatma Amdouni, Boutheina Bessadok, Ghada Tagorti, Saloua Sadok

**Affiliations:** 1 Aquaculture Laboratory, National Institute of Sea Sciences and Technologies, 28 Rue de 2 Mars 1934, Salamboo 2025, Tunisia; fatmaamdouni12@yahoo.fr (F.A.); t.ghada016@gmail.com (G.T.); 2 B3Aqua Laboratory, National Institute of Sea Sciences and Technologies, 28 Rue de 2 Mars 1934, Salamboo 2025, Tunisia; ben.sadok_boutheina@yahoo.fr (B.B.); salwa.sadok@instm.rnrt.tn (S.S.)

**Keywords:** nodavirus, chitosan, gene expression, prevention

## Abstract

**Simple Summary:**

Viral Encephalopathy and Retinopathy (VER) is caused by an RNA virus named *Betanodavirus* representing a major problem in Mediterranean marine aquaculture resulting in great economic losses. Control of VER currently relies on effective biosecurity measures and vaccines. Natural non-toxic biopolymers such as chitosan are produced commercially from crab and shrimp shell waste offering useful biomolecules for a wide range of applications for their antimicrobial and antioxidant activity, immunostimulant effect and bioactive coating. This study evaluates the in-vitro antiviral activity of chitosan extracted from the exo-skeleton of *Parapenaeus longirostris*, focusing on its ability to modulate the innate immune response in E-11 cells challenged with nodavirus as well as its impact on nodavirus RNA2 gene expression. Using qPCR, results reviewed here demonstrated a modulation of the innate immune gene expression and a reduction in virus load suggesting that chitosan was found to be a suppressor of nodaviral infection in fish cell systems.

**Abstract:**

This study explores whether crustacean products inhibit viral infections in aquaculture. Chitosan (CHT) was extracted from waste products of *Parapenaeus longirostris*. Biochemical composition, viscosity measurement, molecular weight, structure and cytotoxicity tests were used to characterize the extracted chitosan. Cultures of E-11 cells derived from snakehead *Ophicephalus striatus* were inoculated with 10^6.74^ TCID_50_ of an isolate of betanodavirus genotype RGNNV (redspotted grouper nervous necrosis virus) after being treated with solutions of 0.3% CHT for 1 h at room temperature. The antiviral effect of CHT was assessed by comparing the ability of RGNVV to replicate and produce cytopathic effects on CHT-treated cell cultures. The change in RNA expression levels of the nodavirus capsid protein gene and three mediator genes in infected cells with or without CHT treatment was evaluated by qPCR. Changes in gene expression compared to control groups were monitored at 6, 24, 48 and 71 h post treatment in all target gene transcripts. The CCR3 expression in CHT treated cells showed a significant increase (*p* < 0.05) until day 3. On the other hand, the expression of TNF-α decreased significantly (*p* < 0.05) in CHT treated cells throughout the experimental period. Likewise, the expression of the IL-10 gene showed a significant downregulation in CHT treated cells at all time points (*p* ≤ 0.05). As further evidence of an antiviral effect, CHT treatment of cells produced a reduction in virus load as measured by a reduced expression of the viral capsid gene and the increase in RQ values from 406 ± 1.9 at hour 1 to 695 ± 3.27 at 72 h post inoculation. Statistical analysis showed that the expression of the viral capsid gene was significantly lower in cells treated with chitosan (*p* ≤ 0.05). These results improve our knowledge about the antiviral activity of this bioactive molecule and highlight its potential use in fish feed industry.

## 1. Introduction

Viral Encephalopathy and Retinopathy (VER) is a major problem in Mediterranean marine aquaculture resulting in great economic losses [1]. VER is caused by an RNA virus belonging to the *Betanodavirus* genus of the family *Nodaviridae* [2]. Its genome is composed of two positive-sense, single-stranded, RNA1 and RNA2 segments coding for the viral RNA polymerase (RdRP) and capsid protein (CP), respectively. Betanodaviruses are classified into four genotypes: Striped jack nervous necrosis virus (SJNNV), Tiger puffer nervous necrosis virus (TPNNV), Barfin flounder nervous necrosis virus (BFNNV) and Redspotted grouper nervous necrosis virus (RGNNV) [2]. Additionally, reassortant isolates harboring mixed RGNNV and SJNNV genomic segments have been reported and represent a new challenge for Mediterranean aquaculture [3,4].

Control of VER currently relies on effective biosecurity measures and vaccines. Various chemicals and drugs have been tested for their ability to hamper nodavirus replication and showed that Betanodavirus tropism involves the mono amine neurotransmitter system [5]. In other studies, nodavirus loads were significantly reduced using Oligonol (lychee fruit extract) applied during early stages of infection, suggesting the drug may interfere with viral attachment to the cell [6]. Ribavirin, an anti-RdRp drug, also inhibited nodavirus infection in vitro [5].

Natural and non-toxic biopolymers such as chitosan are produced commercially from crab and shrimp shell waste. Their unique properties, biodegradability, biocompatibility, low cost and non-toxicity make them useful for a wide range of applications, especially as a marine-based product for their antimicrobial and antioxidant activity, immunostimulant effect and bioactive coating [7]. Chitosan (CHT) is a linear, polycationic heteropolysaccharide [8]. It consists of two monosaccharides, *N*-acetyl-d-glucosamine (2-acetamido-2-deoxy-β-d-glucopyranose, C8H15NO6, molecular weight (MW) = 221.2), the repeat unit of insoluble chitin, and D-glucosamine (2-amino-2-deoxy-β-d-glucopyranose, C6H13NO5, MW = 179.17), linked together by β-(1→4) glycosidic bonds. CHT is produced by alkaline or enzymatic deacetylation from chitin, the structural component of the arthropod exoskeleton and fungal cell wall. CHT possesses a well-documented, broad-spectrum, direct, antimicrobial activity against filamentous fungi, yeasts, Gram positive and Gram-negative bacteria [9,10,11]. Antiviral activity of CHT was ruled out in tobacco necrosis virus (TNV)-inoculated bean leaves [12].

The innate immune system in fish is important for control of viral diseases [13,14] and includes cytokines that regulate the induction of innate immunity [15]. Cytokines include a wide range of molecules such as interferon (IFN), interleukin (IL-1) or tumor necrosis factor (TNF) that can function either as activators or suppressors of immune activity. This study evaluates the in-vitro antiviral activity of chitosan extracted from the exoskeleton of *Parapenaeus longirostris*, focusing on its ability to modulate the gene response in E-11 cells challenged with nodavirus as well as its impact on nodavirus RNA2 gene expression.

## 2. Materials and Methods

### 2.1. Chitin Extraction and Chitosan Production

Byproducts of deep-water rose shrimp *Parapenaeus longirostris* collected from the North coasts of Tunisia were cleaned to recover shells which were dried for subsequent utilization. Chitin was prepared according to the method described by Gopalakannan in 2000 [16]. De-proteinization was performed by heating shrimp shells in a 3% sodium hydroxide (NaOH) solution. The de-proteinized product was then demineralized with a solution of HCl (1.25N). The obtained chitin was dried, ground and preserved from moisture. Chitosan was obtained following deacetylation using NaOH (12.5M) [17]. To 1 g of chitin, 30 mL NaOH was added and the solution incubated during 4 h at 100 °C. The resultant product was thoroughly washed with distilled water and dried at room temperature. Finally, 0.1 g of chitosan was dissolved in 30 mL of 2% acetic acid using a magnetic stirrer at room temperature according to the method of Rinaudo in 2006 [18]. This stock solution was used for subsequent dilution when needed.

### 2.2. Chitin/Chitosan Characterization

#### 2.2.1. Biochemical Composition

Moisture and ash were determined according to the AOAC method [19]. Chitin moisture content was determined by drying sample to a constant weight at 103 ± 2 °C and the ash content was found following incineration in a muffle furnace at 550 °C. Lipid and free carbohydrate were extracted according to the methods of Folch in 1957 [20,21], respectively.

Total nitrogen was determined by Flow Injection Analysis after a sample digestion with concentrated sulfuric acid (H_2_SO_4_) in the presence of a catalyst (H_2_O_2_) and under heat [22]. Protein content was calculated by multiplying the total nitrogen content by 6.25.

#### 2.2.2. Viscosity Measurement and Molecular Weight Determination

The viscosity of the chitosan solution was measured with Ubbelohde viscometer (AVS 470, SI Analytics, Weilheim in Oberbayern, Germany) at 25 °C. The molecular weight of the resulting chitosan was determined using the Mark-Houwink formula [23].

#### 2.2.3. Structural Elucidation

An IRAffinity-1 Fourier Transform Infrared Spectrophotometer (SHIMADZU, Canby, OR, USA) using potassium bromide pellets was used to determine the acetylation degree of chitin and chitosan by the method of Brugnerotto in 2001 [24].

### 2.3. Cell Line and Viral Strain

The fibroblastic clone E-11, derived from the SSN-1 cell line of snakehead fish *Ophiocephalus striatus*, [25] was used to study the cytotoxic and antiviral effects of chitosan. Cells were cultured in 25 cm^2^ flasks at 25 °C in Leibovitz’s L-15 medium (Eurobio, Les Ulis, France), with 10% fetal bovine serum (FBS) and 2% penicillin-streptomycin solution (100 mg/mL streptomycin and 100 IU/mL penicillin). The Tunisian nodavirus strain, RGNNV-131/Aq/10 isolated from European sea bass *Dicentrarchus labrax* [26], was used. Cell culture infections were monitored for cytopathic effect (CPE) and harvested at full CPE seven days post-inoculation. Viral supernatants were clarified by low-speed centrifugation (3000× *g* for 15 min) and stored in aliquots at −80 °C. To titrate the isolate, viral supernatant diluted 10^−1^ to 10^−8^ was used to infect E-11 cells and the TCID_50_/mL calculated by the Spearman-Karber method [27]. Cytopathic effect (CPE) was observed each day and the titer determined on day 7.

### 2.4. In-Vitro Infection of E-11 Cells

Fresh 96 well plates of E-11 cells were treated with different concentrations of solubilized chitosan in acetic acid to obtain 0.3%, 0.5%, 0.7% and 2% solutions. Three modes of treatment were tested. The chitosan was added preventively 1 h before inoculation with nodavirus strain (mode 1), as a curative solution 1 h after infection (mode 2) or mixed and incubated with the virus for 1 h at RT before E-11 inoculation (mode 3). Treated cells (CHT + NNV) were inoculated with 50 µL of the nodavirus preparation of 1.0 × 10^7^ TCID_50_/mL^−1^, which resulted in a MOI of 0.1. Untreated cells (negative control) were mock inoculated with L-15 medium and positive control (NNV only) wells were inoculated with nodavirus suspension only. An additional negative acetic acid control was also included. Cytopathic effects of NNV were examined and photographed using an inverted microscope (Motic AE2). The supernatants from virus inoculated wells, different concentration chitosan treated wells and negative control wells were collected and stored at −80 °C prior to RNA extraction. The cells were harvested in duplicates wells after 6, 24, 48 and 72 h post infection for the assessment of viral and cellular gene expression.

### 2.5. Cytotoxicity Activity Test by MTT Assay

The effect of chitosan on the growth of E-11 cells was tested for 7 days under in vitro conditions using the MTT (3-[4,5-dimethylthiazol-2-yl]-2,5 diphenyl tetrazolium bromide) assay as described by [28]. The test is based on the reduction of soluble yellow MTT tetrazolium salt to a blue insoluble MTT formazan product by mitochondrial succinic dehydrogenase. After 24 h of exposure, the test medium was replaced by 10 μL of 5 mg/mL MTT (Sigma-Aldritch, Burlington, MA, USA) in PBS. After incubation for 4 h at 25 °C, the staining solution was carefully removed by aspiration and the cells were rinsed twice with PBS, and 200 μL/well of DMSO was added to solubilize the blue formazan crystals produced. The absorbance of each well was measured at 590 nm (test wavelength) in the microplate reader (Fluostar Optima, BMG Labtech, Ortenberg, Germany).

### 2.6. RNA Extraction

RNeasy Mini Kit (Qiagen, Germantown, MD, USA) was used to purify total RNA from cell supernatants according to the manufacturer’s instructions. RNA elution was performed in a final volume of 30 µL of RNase-free water before storage at −80 °C. The concentration of the isolated RNA was measured using Nanodrop 2000 (Thermo scientific, Waltham, MA, USA). Nucleic acid concentration was obtained directly in terms of ng/µL and the 260:280 ratios used to estimate of the purity of the isolated RNA.

### 2.7. Relative Quantification of Target Genes by ΔΔCT 

Random hexamers were used to synthesize the first strand cDNA using the SuperScript first strand synthesis system for RT-PCR (Invitrogen, Waltham, MA, USA). The extracted RNA (50 ng–1 µg) was mixed with 1 µL of random hexamers (50 ng/µL) and 1 µL of 10 mM dNTP mix. This mixture was incubated at 65 °C for 10 min and subsequently placed at 4 °C for at least 1 min. This reaction was then completed with the following reagents: 200 U of the reverse transcriptase (SuperScript III, Thermo Fisher Scientific, Waltham, MA, USA), 40 U of RNase OUT, 2 µL of 10× first strand buffer, 2 µL of 0.1 M DTT and 4 µL of 25 mM MgCl_2_. This mixture was incubated in an automatic thermal cycler (BioRad, Hercules, CA, USA) at 50 °C for 50 min followed by 5 min at 85 °C. The remaining RNA was eliminated with 0.5 µL of RNase H (2 U/µL) at 37 °C for 20 min. The 7500 Real Time PCR System (Applied Biosystems, Waltham, MA, USA) and the sets of primers used for the viral and immune gene expression assays are published by [29,30], respectively. For the PCR reaction, cDNA was added to a pre-cooled mix containing 8 µL of RNAase free water, 1 µL of each primer (20 µM) and 12 µL of the iQ SYBR Green Supermix (BioRad, Hercules, CA, USA), which includes the iTaq DNA polymerase. After an activation step at 95 °C for 3 min, 40 PCR cycles composed of a denaturation step of 15 s at 94 °C, 30 s of annealing at 58 °C and 30 s of extension at 72 °C were performed. The specificity was determined by a first-derivative melt curve. Two negative controls using diethylpyrocarbonate (DEPC)-treated water as template (no RNA template) were included within each experiment. In addition, nodavirus free samples were first tested with the present protocol and found to confirm the specificity of the primers (data not shown). Each sample was analyzed in duplicate and C_T_ values were plotted against the logarithm of the amount of template and a linear regression was performed. Normalization of the results was applied using the ratio between the mean C_T_ values obtained with the target gene primers and those obtained with the β-actin primers and a calibrator gene (before treatment). RNA quantities were estimated by the application of the formula of Livak in 2001 [31]:

Δ CT (target gene) = CT (target gene) − CT (β-actin)

Δ CT (calibrator) = CT (calibrator) − CT (β-actin)

ΔΔCT (target gene) = Δ CT (target gene) − Δ CT (calibrator)

RQ (target gene) = 2^−ΔΔ*C*^_T (target gene)_

(*) Target gene: Viral or immune gene targeted after chitosan treatment, (**) Calibrator: Viral or immune gene targeted before chitosan treatment.

### 2.8. Statistical Analysis

Differences in the response between infected and mock groups and differences between samples from wells with and without CHT were represented as means ± SD. Data were analyzed by one-way analysis of variance (ANOVA) using Statistical Package for Social Sciences Software (SPSS) version 20.0 (SPSS Inc., Chicago, IL, USA) and when statistically significant differences were observed (*p* < 0.05) a comparison of mean test was applied.

## 3. Results

### 3.1. Chitin and Chitosan Characterization

**Figure 1:**
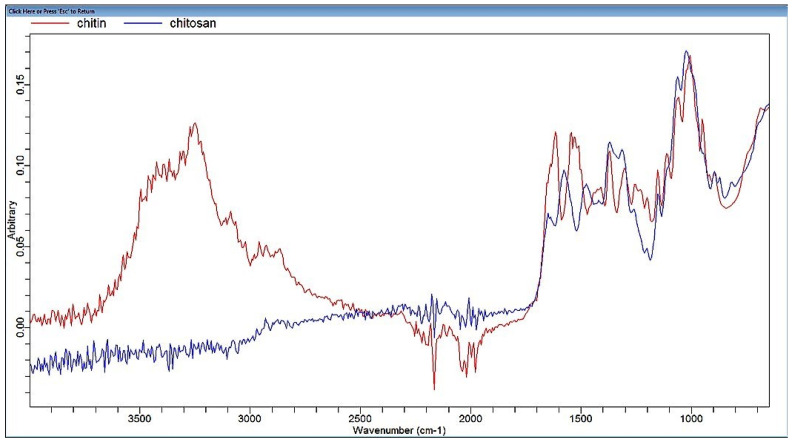
Infrared spectrum (FTIR) of chitin and chitosan extracted from *Parapenaeus longirostris* waste.

Chitin and its derivatives are versatile biomolecules of great potential biological activity [32,33]. The total residual protein content of chitin extracted from *P. longirostrus* was comparable to that found in commercial chitin (1.53 ± 0.05 g/100 g dw chitin), indicating the good deproteination of the raw material. Surprisingly, a substantial quantity of lipid was found in chitin (Table 1) which may be related to the presence of lipoproteins that would protect them from hydrolysis during extraction [34]. A low level of free carbohydrate was detected in the supernatant. Shrimp shell demineralization is an important step in the extraction process of chitin. The latter is considered of good quality when its ash content is <1% [35,36]. In this study, chitin ash level was lower than the recommended threshold (Table 1). As an edible additive, a recommended moisture value has been established for high quality products such as chitin. Here, *P. longirostrus* chitin moisture (Table 1) was significantly lower than the threshold standard (10%) [36]. The degree of acetylation (DA) is an essential feature of chitin and chitosan as it shows the percentage of acetylated units relative to the total number of units and can be measured using Fourier Transform Infrared Spectroscopy by identifying the different functional groups absorbing at constant frequencies existing in the products (Figure 1). Table 2 shows the significantly higher DA in chitin when compared to chitosan. Such results conform to established attributes where a sample with a degree of acetylation ≤30% is referred to chitosan [37]. The DA influences the solubility, interaction between chains, flexibility, conformation and consequently their fields of application [38]. The viscosity and the molecular mass of chitin and chitosan are illustrated in Table 2. The viscosity of chitosan solution is lower than that of chitin as it depends on several parameters such as the deacetylation process and the raw material [39]. Such parameters also affect their molecular weight.

### 3.2. Effect of Chitosan Concentrations on Cell Viability

The cytotoxic property of chitosan on the E-11 cell line was evaluated via the MTT test. Table 3 clearly shows that cell survival for 3 days without treatment was high (86%). The cell treatment with 2% AA alone caused a critical reduction in viability to approximately 68%.

The presence of 0.3% CHT in 2% AA did not reduce viability further, so the 0.3% CHT has no impact on cell viability beyond that of the 2% AA it is dissolved in. A concentration of 0.3% of chitosan inhibited cell viability to approximately 67.99% at the end of 3 days (Table 3) but did not alter the morphological appearance of the monolayer. The increase in CHT concentrations was correlated with the increase in cell cytotoxicity, indeed, higher concentrations of CHT did reduce viability further, to as low as 41% for 1% CHT. Thus, CHT at 0.3% was chosen as the best concentration to perform the rest of the tests.

### 3.3. Effect of Chitosan Treatment on Cytopathic Effect (CPE) after Nodavirus Infection

**Figure 2:**
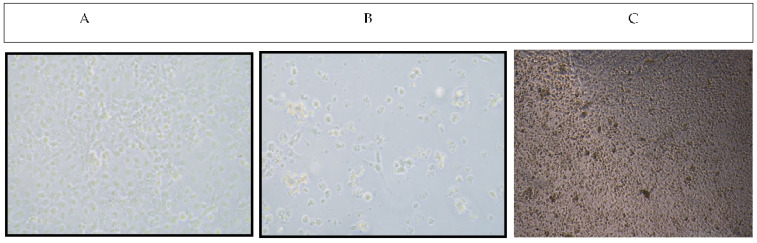
Susceptibility of E-11 cells to nodavirus and chitosan treatment (**A**) Confluent uninfected E-11 cells at day 4 post-seeding. (**B**) Extensive CPE with multiple vacuolation in E-11 cells infected with nodavirus at day 4 post inoculation. (**C**) Nodavirus infected E-11 cells treated with 0.3% Chitosan at day 4 post inoculation.

In nodavirus infected wells, CPE with typical multiple vacuolation was observed in cells after 3 to 4 dpi (Figure 2B). Initially, the specific CPE developed as localized areas of rounded and refractile cells that later spread over the monolayer to form a network of degenerating cells. The monolayer was completely disintegrated after 7 days. No CPE was observed in mock cells (Figure 2A). Chitosan at 0.3% was added preventively 1-h before inoculation with nodavirus strain (mode 1). As demonstrated in Figure 2C, we observed that E-11 treated wells with CHT showed a clear inhibition of nodavirus proliferation (Figure 2C).

### 3.4. Effect of Chitosan Treatment Modes on Nodavirus RNA2 Replication

**Figure 3:**
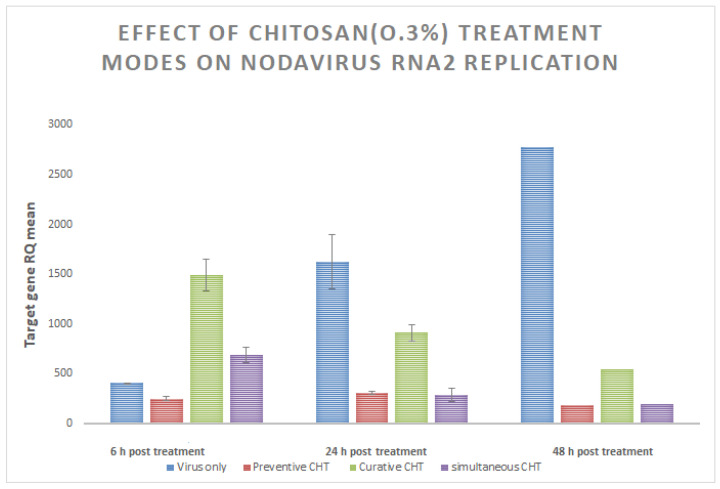
Expression of nodavirus capsid protein (CP) gene in E-11 at different times points (6, 24 and 48 hours post treatment) following treatment of Chitosan (CHT) at 0.3%. Three different modes were tested: preventive mode (mode 1: red bars), curative mode (mode 2: green bars) and simultaneous mode (mode 3: purple bars) for the evaluation of the chitosan effect on nodavirus RNA replication. Nodavirus only inoculated cells are shown in blue bars. Fold expression was calculated as 2^−∆∆Ct^. Control group (no CHT treatment) was used as the calibrator.

Three different modes were tested for the evaluation of the chitosan effect on nodavirus RNA replication in E-11 cells as described above. Supplementary Table S1 shows detailed results obtained when amplifying nodavirus RNA2 from cells that received a pre-treatment of CHT 0.3% for 1 h followed by nodavirus inoculation at MOI = 0.1 (mode 1), cells that were inoculated by nodavirus (MOI = 0.1) and incubated for 1 h then CHT 0.3% was added (mode 2) and finally from cells inoculated by a mixture of CHT 0.3% and nodavirus incubated for 1 h at room temperature (mode 3). The use of chitosan as preventive mode (mode 1) resulted in the best inhibition of nodavirus CP gene replication (Figure 3). However, both mode 2 and mode 3 have resulted in a nodavirus inhibition as well compared to positive control well (Supplementary Table S1) suggesting that the molecules of this polymer can act on extracellular (plasma membrane) and/or intracellular level (penetration of chitosan into the E-11cell). Thus, it is necessary to carry out more studies about the biological activity of these molecules to propose better control strategies of in-vitro nodavirus inhibition.

### 3.5. Effect of Chitosan Concentrations on Nodavirus RNA2 Replication

**Figure 4:**
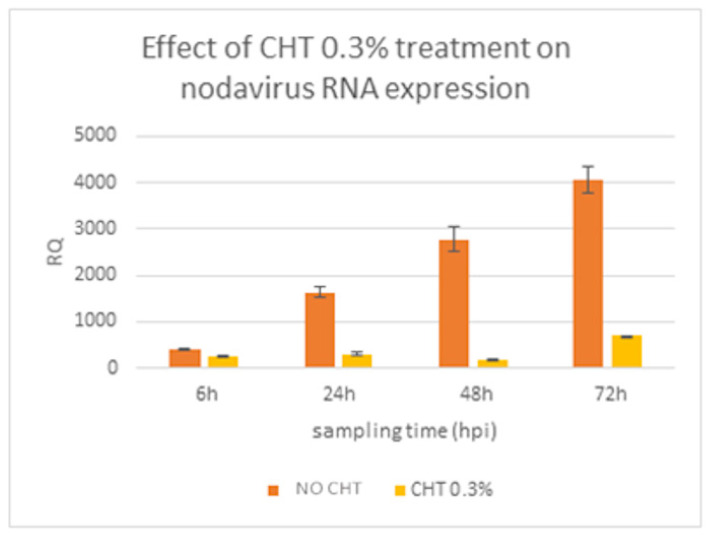
Expression of nodavirus capsid protein (CP) gene in the E-11 cell line with RGNNV nodavirus and expression of nodavirus capsid protein gene in E-11 cells treated with 0.3% Chitosan (CHT) at different hours post inoculation. Nodavirus only inoculated wells are shown in dark orange while inoculated cell previously treated with 0.3% of chitosan (CHT) are in yellow. Fold expression was calculated as 2^−∆∆Ct^. Control group (0 h post challenge) was used as the calibrator.

Three inoculation modes were tested in the present study, however, only the results for use of CHT as a preventive treatment (mode 1) will be discussed here as this gave the best inhibitory activity of the viral capsid protein gene expression (Supplementary Table S1). Supplementary Table S2 presents the results of the viral capsid protein expression levels before and after CHT treatment at various CHT concentrations on E-11. Figure 4 shows that the expression of the viral gene in control cell cultures with no CHT treatment (orange bars) cells. This shows a CHT concentration of 0.3% provides optimal inhibition of viral RNA. Nodavirus capsid gene expression level increased steadily over the 72 h post inoculation, demonstrating that viral replication took place. On the other hand, and as further evidence of an antiviral effect, CHT treatment of cells produced a reduction in virus load as measured by a subexpression of the viral capsid gene and the increase in RQ values from 406 ± 1.9 at hour 1 to 695 ± 3.27 at 72 h post inoculation. Statistical analysis showed that the expression of the viral capsid gene was significantly lower in cells treated with chitosan (*p* ≤ 0.05). This indicates that the presence of 0.3% CHT caused major differences in the transcription of the nodavirus RNA2 gene and was effective in inhibiting the replication of a key nodavirus protein in E-11 cells.

### 3.6. Expression Level Changes in Cell Mediator Genes in Response to Virus Infection and Chitosan Treatment

**Figure 5:**
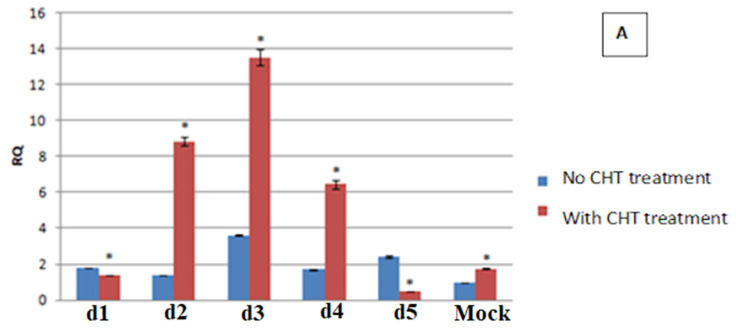

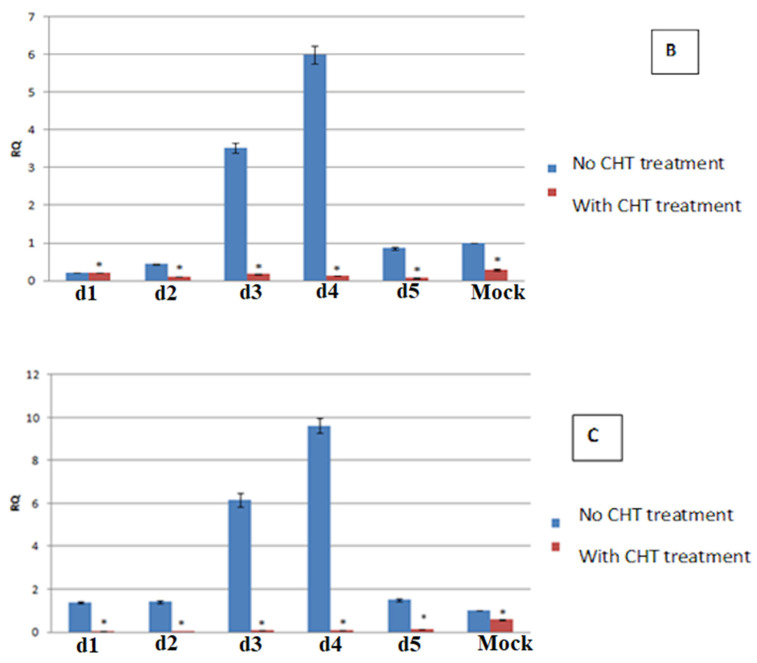
Expression analysis of three cellular genes in the E-11 cell line treated with 0.3% Chitosan for different time periods (d, days post challenge). (**A**) Relative qualitification (RQ) values for the CCR3 gene transcript, (**B**) Relative qualitification (RQ) values for the TNF-α gene transcript and (**C**) Relative qualitification (RQ) values for the Il-10 gene transcript. Cultures shown in presence of chitosan (CHT) treatment (column in red with * sign) and without chitosan (CHT) treatment (column in blue). Fold expression was calculated as 2^−∆∆Ct^. Control group (0 h post challenge) was taken as the calibrator.

The gene expression patterns of CCR3, IL-10 and TNFα in E-11 cells is depicted in Figure 5 where changes with respect to control groups with no CHT treatment were observed in all three target gene transcripts. The CCR3 expression in CHT treated cells (preventive mode) showed significant increase over non-treated controls (*p* < 0.05) until day 3 but then decreased tenfold on the 5th day (Figure 5A). On the other hand, the expression of TNF-α decreased significantly due to CHT treatment (*p* < 0.05) throughout the experimental period, as shown in Figure 5B. The expression of IL-10 also was reduced by CHT treatment on day 1 and at subsequent time points. Figure 5C shows a significant downregulation for the IL-10 gene following the inoculation of virus onto 0.3% chitosan treated E-11 cells (*p* ≤ 0.05).

## 4. Discussion

Due to its importance for aquaculture, sea bass rearing is continuously expanding and improving, mostly in southern European and North African countries. Generally, vaccination is one of the best strategies in disease management that has been used to reduce the antibiotic use in fish production and to prevent disease occurrence. To date, only two commercially available vaccines which are based on inactivated RGNNV nodavirus exist on the market, however, a multitude of different types of vaccine for VNN have been tested in the laboratory such as recombinant protein, formalin inactivated betanodavirus, DNA-based vaccines and virus-like particle vaccines [39,40]. Thus, the stimulation of the host’s defense mechanisms by elicitors may represent an effective strategy as well. Studies have shown that chitin and chitosan can act as potent immune stimulators in fish and shellfish. Indeed, chitosan enhanced fish survival rate and immune response, increasing IgM content, lysozyme activity and mRNA levels of interleukin (IL)-1β, IL-2 and interferon (IFN)-γ2 [40,41]. Additionally, an oral chitosan-encapsulated DNA vaccine (CP-pNNV) for the nodavirus up-regulated the expression of genes related to the cell-mediated cytotoxicity (CMC; tcrb and cd8a) and the interferon pathway (IFN; ifn, mx and ifng) [42].

During virus infection, capsids are typically responsible for attachment of a virus to the host cell prior to subsequent release of the genome for replication. Here we suggest that a preventive chitosan treatment of E-11 cells can block nodavirus replication. This can be due to different methods. Our results show that chitosan can inhibit RGNNV nodavirus in-vitro replication proper, however, it is still unknown which stage of replication is the target of chitosan. Work carried out by [42,43] showed that adding chitosan to a culture after phage adsorption is as effective as adding chitosan before inoculation. Due to this, it seems improbable that chitosan inhibits infection by preventing phage adsorption.

Innate defenses against pathogens are the first line of defense against the non-self and are comprised of a complex network of molecules and cells that operate to rapidly kill and/or inactivate the putative pathogen. The current status of knowledge on sea bass immune system has been reviewed [44] and it is known that both cellular and humoral immunity work cooperatively to control nodavirus infection. Fish cytokines are involved in several steps of the immune response and can be divided into interferons (IFNs), interleukins (ILs), tumor necrosis factors (TNFs), colony stimulating factors and chemokines [45]. In the present study, IL-10, TNFα and CCR3 receptor gene transcription levels were assessed following chitosan treatment of E-11 cells infected with nodavirus. Results showed that the transcription of RNA encoding the fish cell chemokine receptor (CCR3) gene was up regulated after the chitosan treatment which induced its transcription. Not many CCR sequences have been published from teleosts. In rainbow trout and other fish, homologues for CCR3 and CCR9 have been isolated [46] and analysis showed their expression was significantly upregulated after infection with *V*. *anguillarum.* This positive induction may initiate defense responses in the fish cell and contribute to the accumulation and activation of eosinophils and other inflammatory cells at the viral infection site regulating the immune cell migration. Our results are in accordance with work carried out by [47] showing that in the context of an experimental infection with nodavirus, all the CC chemokines studied were significantly induced in the brain, suggesting an important role for these chemokines in the recruitment of leukocytes to major replication sites.

TNFα (tumor necrosis factor alpha) is a pro-inflammatory cytokine that plays an important role in cell proliferation, differentiation, necrosis, apoptosis and induction of other cytokines. TNFα has been identified, cloned and characterized in several bony fish [4]. The most interesting difference between fish and mammal TNFα concerns the weak in vitro effects of TNFα on phagocyte activation in several bony fish [48]. Un-expectedly, its expression was steadily reduced after virus infection in the presence of chitosan treatment in the present work. In addition, chitosan provoked strong suppressive effects on the transcription of the IL-10 gene coding for an anti-inflammatory cytokine that acts as a suppressor of inflammatory responses. This suggests its probable key role in the inhibition of viral mRNA transcription as described by [49]. The ability of chitosan to induce interferon synthesis can be an additional important factor in the antiviral activity observed post CHT treatment of E-11 cells. 

## 5. Conclusions

The data reviewed here suggest that chitosan is a suppressor of nodaviral infection in fish cell systems.

Further studies including the determination of infectious virus titer at different time points and in vivo challenge studies will be highly beneficial.

## Figures and Tables

**Table 1:** Biochemical composition of chitin extracted from *Parapenaeus longirostris* caught from the north of Tunisia. Values (g/100 g) are reported as dry mass (*n* = 3 in each analysis, ±: Standard Error).

Parameters	Chitin
Moisture	4.16 ± 0.16
Ash	0.51 ± 0.05
Lipid	2.63 ± 0.16
Carbohydrate	0.025
Total nitrogen	0.19 ± 0.01
Total protein	1.18 ± 0.32

**Table 2:** Physical proprieties of chitin and chitosan extracted from *Parapenaeus longirostris* caught from the north of Tunisia. Values (g/100 g) are reported as dry mass (±: Standard Error).

Parameters	Chitin	Chitosan
DA (%)	87.08	25.46
Molecular weight (KDa)	372.58 ± 1.4	10.71 ± 0.33
Viscosity (dL/g)	14.91 ± 0.06	10.13 ± 0.32

DA (%): Percentage of the acetylation degree.

**Table 3:** E-11 viability test using MTT. The table includes viability percentages (%) of E-11 cells mock treated mock with L-15 medium only, E-11 cell treated with 2% acetic acid (AA) and E-11 cells treated with chitosan (CHT) dissolved in 2% AA at different concentration (0.3%, 0.5%, 0.7% and 1%).

Parameter	Cells Only	Cells + AA	Cells + CHT 0.3%	Cells + CHT 0.5%	Cells + CHT 0.7%	Cells + CHT 1%
OD	696	668	534	426	463	324
OD	619	690	496	482	391	293
OD mean	657.5	679	515	454	427	308.5
Cell viability %	86.79	67.98	67.99	59.93	56.37	40.73

OD: Optic density.

## Data Availability

The data that support the findings of this study are available on request from the corresponding author. Some of the data are not publicly available due to the privacy or ethical restrictions.

## Supplementary Materials

The following are available online at https://www.mdpi.com/article/10.3390/ani11113097/s1, Table S1: Modulation of Nodavirus RNA expression levels impacted by chitosan (0.3%) treatment mode: preventive (mode 1), curative (mode 2) or incubated mixed solutions (mode 3) at different sampling points. Table S2: Modulation of Nodavirus RNA expression levels impacted by preventive chitosan E-11 cell treatments at different concentrations and sampling points.
