# Supplementary material for: Chitosan Treatment of E-11 Cells Modulates Transcription of Nonspecific Immune Genes and Reduces Nodavirus Capsid Protein Gene Expression
Source: Animals (Basel). 2021 Oct 29;11(11):3097. doi: 10.3390/ani11113097 (PMC8614572; doi:10.3390/ani11113097)
Supplement: Supplementary file 1 [file animals-11-03097-s001.zip › animals-1312871-supplementary.pdf]

Table S1. Modulation of Nodavirus RNA expression levels impacted by chitosan (0.3%) treatment mode: preventive (mode 1), curative (mode 2) or incubated mixed solutions (mode 3) at different sampling points. UN: undefined value. Cal gene: calibrator gene. *The expression profiles of nodavirus RNA2 was studied using qPCR and the data presented as the average of the relative quantification (RQ) of the target genes after normalization by the reference gene β-actin ± SD. The β-actin gene showed the lowest Ct value changes compared to other housekeeping genes and had a specific melting peak (data not shown). Hence, this gene was used as the internal control. The fold change in expression of the target genes in each of the sample groups were determined with respect to the calibrator gene at each time point.*

Sampling time	Ct viral RNA	Ct b-actin RNA	ΔCT	ΔΔCT	RQ	Mean	SD
Inoculation of E-11 cell with RGNNV nodavirus only							
6 hpi	18.90	18.83	0.067	-8.66	405.42	406.83	1.99
	18.79	18.742	0.057	-8.67	408.24		
24 hpi	18.98	20.99	-2.00	-10.73	1706.71	1628.75	110.24
	19.00	20.87	-1.86	-10.59	1550.8		
48 hpi	18.94	21.75	-2.804	-11.53	2965.99	2774.02	271.49
	18.99	21.6	-2.604	-11.33	2582.05		
72 hpi	18.97	22.163	-3.186	-11.91	3865.13	4059.11	274.34
	18.90	22.226	-3.324	-12.05	4253.1		
Cal gene	32.99	24.26	8.73	0	1	UN	UN
Inoculation of RGNNV nodavirus on E-11 treated with CHT at 0.3%: Preventive mode (mode 1)							
6 hpi	27.28	26.447	0.842	-7.8883	236.927	251.09	20.03
	27.25	26.578	0.679	-8.0513	265.267		
24 hpi	26.25	25.946	0.306	-8.4238	343.413	310.76	46.17
	26.51	25.907	0.610	-8.1195	278.108		
48 hpi	27.41	26.107	1.306	-7.4239	171.718	184.45	18.00
	27.21	26.110	1.106	-7.6234	197.184		
72 hpi	33.83	34.539	-0.70	-9.4375	693.379	695.69	3.27
	33.83	34.548	-0.71	-9.4471	698.008		
Cal gene	32.99	24.260	8.730	0	1	UN	UN
Inoculation of RGNNV nodavirus on E-11 treated with CHT at 0.3%: Curative mode (mode 2)							
6 hpi	24.63	26.447	-1.812	-10.54	1491.24	1381.36	155.39
	24.99	26.578	-1.582	-10.312	1271.48		
24 hpi	24.25	25.9465	-1.693	-10.423	1373.6512	912.71	651.85
	25.81	25.9072	-0.089	-8.8195	451.78732		
48 hpi	25.61	26.1076	-0.493	-9.2239	597.95785	541.74	79.50
	25.91	26.1101	-0.193	-8.9234	485.52440		
72 hpi	26.83	34.5392	-7.707	-16.437	88752.49	122696.61	48004.24
	26.02	34.5488	-8.526	-17.257	156640.74		
Cal gene	32.99	24.2607	8.730	0	1	UN	UN
Inoculation of RGNNV nodavirus on E-11 treated with CHT at 0.3%: Mixed mode (mode 3)							
6 hpi	25.63	26.447	-0.812	-9.5423	745.62	690.68	77.69
	25.99	26.578	-0.582	-9.3123	635.74		
24 hpi	26.25	25.9465	0.306	-8.4238	343.41	284.65	83.09
	26.81	25.9072	0.910	-7.8195	225.89		
48 hpi	27.61	26.1076	1.506	-7.2239	149.48	196.12583	65.95
	26.91	26.1101	0.806	-7.9234	242.76		
72 hpi	27.83	34.5392	-6.707	-15.437	44376.24	46294.045	2712.17
	27.72	34.5488	-6.826	-15.557	48211.8		
Cal gene	32.99	24.2607	8.730	0	1	UN	UN

Table S2. Modulation of Nodavirus RNA expression levels impacted by preventive chitosan E-11 cell treatments at different concentrations and sampling points.

Sampling time	Ct viral RNA	Ct b-actin RNA	ΔCT	ΔΔCT	RQ	Mean	SD
Inoculation of E-11 cell with RGNNV nodavirus only							
6 hpi	18.90	18.83	0.067	-8.66	405.42	406.83	1.99
	18.79	18.742	0.057	-8.67	408.24		
24 hpi	18.98	20.99	-2.00	-10.73	1706.71	1628.75	110.24
	19.00	20.87	-1.86	-10.59	1550.8		
48 hpi	18.94	21.75	-2.804	-11.53	2965.99	2774.02	271.49
	18.99	21.6	-2.604	-11.33	2582.05		
72 hpi	18.97	22.163	-3.186	-11.91	3865.13	4059.11	274.34
	18.90	22.226	-3.324	-12.05	4253.1		
Cal gene	32.99	24.26	8.73	0	1	UN	UN
Inoculation of RGNNV nodavirus on E-11 treated with CHT at 0.3%							
6 hpi	27.28	26.447	0.842	-7.8883	236.927	251.09	20.03
	27.25	26.578	0.679	-8.0513	265.267		
24 hpi	26.25	25.946	0.306	-8.4238	343.413	310.76	46.17
	26.51	25.907	0.610	-8.1195	278.108		
48 hpi	27.41	26.107	1.306	-7.4239	171.718	184.45	18.00
	27.21	26.110	1.106	-7.6234	197.184		
72 hpi	33.83	34.539	-0.70	-9.4375	693.379	695.69	3.27
	33.83	34.548	-0.71	-9.4471	698.008		
Cal gene	32.99	24.260	8.730	0	1	UN	UN
Inoculation of RGNNV nodavirus on E-11 treated with CHT at 0.5%							
6 hpi	25.06	25.037	0.03	-8.70	415.95	464.92	69.24
	25.05	25.332	-0.275	-9.00	513.88		
24 hpi	24.99	25.401	-0.405	-9.13	562.34	520.73	58.83
	24.03	24.205	-0.174	-8.90	479.13		
48 hpi	22.51	25.701	-3.187	-11.91	3867.80	2757.21	1570.60
	23.00	24.956	-1.955	-10.68	1646.6292		
72 hpi	22.47	26.069	-3.593	-12.32	5124.87	9000.00	5480.26
	22.47	27.401	-4.922	-13.65	12875.13		
Cal gene	32.99	24.260	8.730	0	1	UN	UN
Inoculation of RGNNV nodavirus on E-11 treated with CHT at 0.7%							
6 hpi	22.38	21.57	0.812	-7.91	241.90554	328.50	122.46
	22.04	22.01	0.033	-8.697	415.09		
24 hpi	22.99	22.121	0.875	-7.85	231.56	280.55	69.27
	23.02	22.655	0.366	-8.36	329.53		
48 hpi	26.74	24.501	2.239	-6.49	89.96	177.98	124.47
	26.53	25.856	0.675	-8.05	266.00		
72 hpi	27.22	26.469	0.757	-7.97	251.30	351.98	142.382486
	27.00	27.101	-0.092	-8.82	452.66		
Cal gene	32.99	24.260	8.730	0	1	UN	UN
Inoculation of RGNNV nodavirus on E-11 treated with CHT at 1%							
6 hpi	22.08	22.057	0.025	-8.70	417.40	428.99	16.39
	22.05	22.11	-0.053	-8.78	440.59		
24 hpi	23.00	23.01	-0.004	-8.73	425.87	431.25	7.60
	23.01	23.05	-0.04	-8.770	436.63		
48 hpi	29.57	29.501	0.073	-8.65	403.74	422.47	26.48

	29.501	29.556	-0.055	-8.78	441.20		
72 hpi	27.446	27.369	0.077	-8.65	402.62	418.42	22.34
	27.369	27.401	-0.032	-8.76	434.22		
Cal gene	32.991	24.2607	8.7303	0	1	UN	UN
